# Effect of microtrichia on the interlocking mechanism in the Asian ladybeetle, *Harmonia axyridis* (Coleoptera: Coccinellidae)

**DOI:** 10.3762/bjnano.9.75

**Published:** 2018-03-06

**Authors:** Jiyu Sun, Chao Liu, Bharat Bhushan, Wei Wu, Jin Tong

**Affiliations:** 1 Key Laboratory of Bionic Engineering (Ministry of Education, China), Jilin University, Changchun, 130022, P.R. China; 2 Nanoprobe Laboratory for Bio- & Nanotechnology and Biomimetics (NLB2), The Ohio State University, 201 W. 19th Avenue, Columbus, OH 43210-1142, USA

**Keywords:** anti-wetting, folding process, interlocking mechanism, micro air vehicles, microtrichia

## Abstract

The hindwings of beetles are folded under the elytra when they are at rest but are extended during flight, which can provide bioinspiration for the design of deployable micro air vehicles (MAVs). Beetle hindwings must be able to be both securely locked under the elytra and freely extended for flight, depending on the required action. To investigate the locking mechanism, this study used environmental scanning electron microscopy (ESEM) to examine the microstructures of the elytra, hindwings and abdomen of the Asian ladybeetle, *Harmonia axyridis* (Pallas, 1773). On the ventral side (VS) of the elytra, the microtrichia show a transitional structure from the lateral edge to the medial edge. On the hindwing surface, the folded regions were observed on both the dorsal side (DS) and the VS. On the abdomen, the microtrichia between the abdominal segments show a cyclical change from sparse to dense in each segment in the middle of the abdomen. Furthermore, the different directions of microtrichia that lead to self-locking friction on the surfaces of the hindwing, elytron and abdomen appear to facilitate interlocking. A model for the interlocking of the hindwings of the *H. axyridis* was established, and its underlying mechanism is discussed.

## Introduction

Insect wings have many properties, such as lightness, thinness, high flexibility and high load capacity [1], as seen in dragonfly wings, for example, that provide inspiration for the design and manufacture of micro air vehicles (MAVs). As a widely distributed order, Coleoptera includes species with various types of complex systems, and the hindwings of beetles are a highly developed deployable structure [2–3]. The folding and self-locking function of beetle hindwings provides new ideas for the design of MAVs, which will help simplify the design of folding wings. Beetle hindwings fold under the forewings (elytra). One advantage of this is size reduction, and another is that membranous hindwings can be protected by the hard elytra. Various parts of the wings play important roles during wing folding in Coleoptera. In beetles, there is an interlocking system composed of the elytra and the thorax [4]. When the thorax and abdomen are interlocked, the forewings provide a complete cover for the hindwings and abdomen [5], and the body forms a stable whole. The successive evolution of interlocking mechanisms and forewing design resulted from selective pressure in strengthening and protective functions [6]. Various hindwing locking mechanisms have been found in beetles [7]. Beetle hindwing folding mechanisms are affected by many factors, including muscular [8], hydraulic [9], and elastic [10–11] properties. Some folding characteristics can also be described according to flexagon (or origami-type) folding in which all nodes conform to four angles, with an odd number of concave or convex folds [12–13]. Contact with the elytra and the abdomen [14] and the blocking of hindwings as the elytra closes in vivo also affect folding [15–16]. The extremely complicated wing-to-body articulation requires direct and indirect wing muscles [8].

In most flying beetles, fields of microtrichia are the predominant wing-locking devices [17]. These microtrichia differ in density, shape, and directionality [5,18–19]. The microtrichia on the thorax, elytra and abdomen of *Ulomoides dermestoides* were studied using SEM, and their structures and distributions were investigated [20]. The possible functions of the microtrichia include producing stridulations through interactions with the elytra, thorax and hindwings; increasing the friction that allows the parts to integrate into a whole; creating a buffer to protect the body from damage; and sensing the surrounding environment [21].

It remains unclear how beetles package their hindwings during folding into the tight space between the elytra and the abdomen. Improving our understanding of the directional friction locks that enable linkage between folded wing layers and between wings, movable elytra and the abdomen and of the morphology of microtrichia (length, width, distance between microtrichia, and direction) will help clarify this folding process. In this study, the morphology and distribution of the microtrichia on the hindwings, elytra and abdomen were investigated in the Asian ladybeetle, *Harmonia axyridis* (Pallas, 1773). Then, an interlocking model describing the folding process of *H. axyridis* hindwings was established. Finally, the anti-wetting function of the hindwings was investigated.

## Materials and Methods

### Beetles

**Figure 1:**
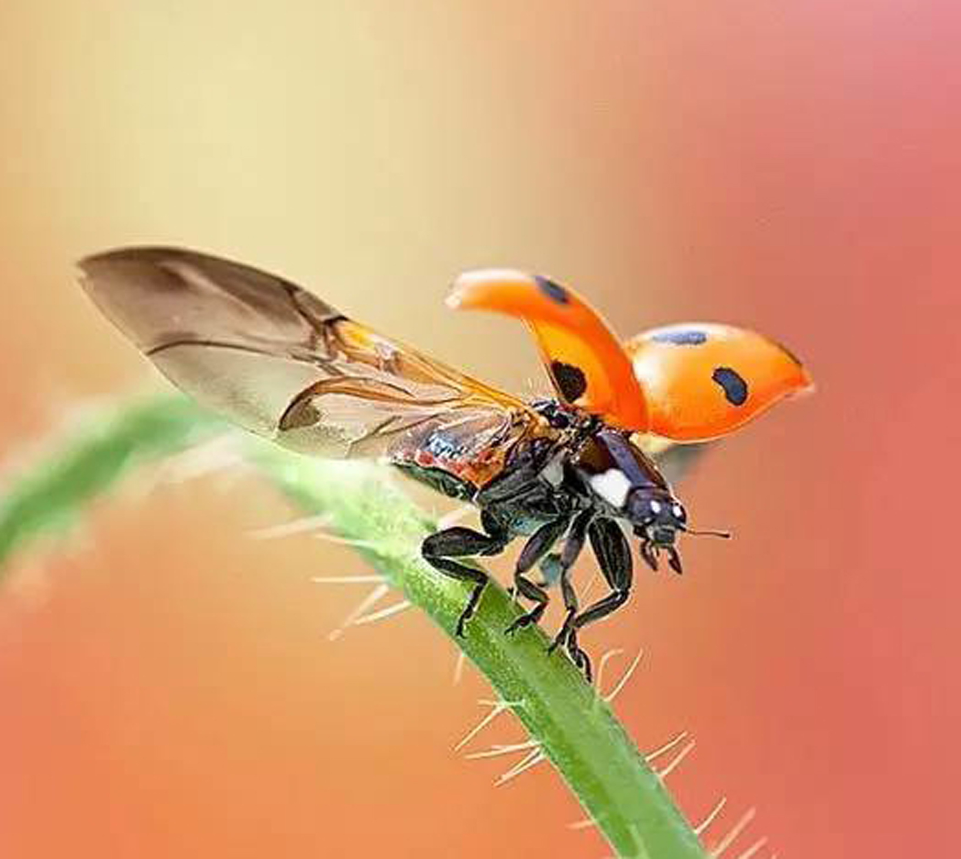
The moment of unfolding of *H. axyridis* hindwings.

Twelve Asian ladybeetles, *H. axyridis*, collected from Changchun, Jilin Province, China (Figure 1) were captured for use in this study. This species is 6–7 mm long and 5–6 mm wide. All elytra and hindwings used for experimental measurements were removed from freshly anaesthetized beetles.

### Wing morphologies

To obtain *H. axyridis* whole-hindwing morphological images, a stereomicroscope (SteREO Discovery V20, Carl Zeiss Microimaging Inc., Germany) was used, and an environmental scanning electron microscope (ESEM) (JEOL JSM-6700F, FEI Company, USA) was used to investigate the morphology of microtrichia on hindwings.

To obtain complete morphological images of elytra and the abdomen, a scanning electron microscope (SEM) (Model EVO-18, Carl Zeiss Microimaging Inc., Germany) was used.

To analyze the hindwing, the wings were first removed from the body and rinsed using distilled water. Before microscopy, the 12 right hindwings collected were divided into three groups. The 6 hindwings in the first group were used to observe surface microtrichia. The hindwings in the second and third groups (3 each) were soaked in an insulated cup filled with liquid nitrogen for 30 s. The hindwings in the second group were cut along their principal transverse folds, and the hindwings in the third group were cut along their main anterior veins. After preparation, the wings were coated with gold and examined using ESEM at an accelerating voltage of 15 kV. Structures were determined from digital pictures by using image analysis software.

The elytra were also removed fresh from the body and were cut into small sections and cleaned with anhydrous ethanol. Then, the elytra were also coated with a thin layer of gold and examined with ESEM The entire abdomen was cleaned with anhydrous ethanol after it was removed from the rest of the body, after which images were captured by ESEM. Wing folding by *H. axyridis* was photographed with a high-speed camera (Phantom V711, Vision Research Inc., USA) at 300 frames/s.

### Contact angle

**Figure 2:**
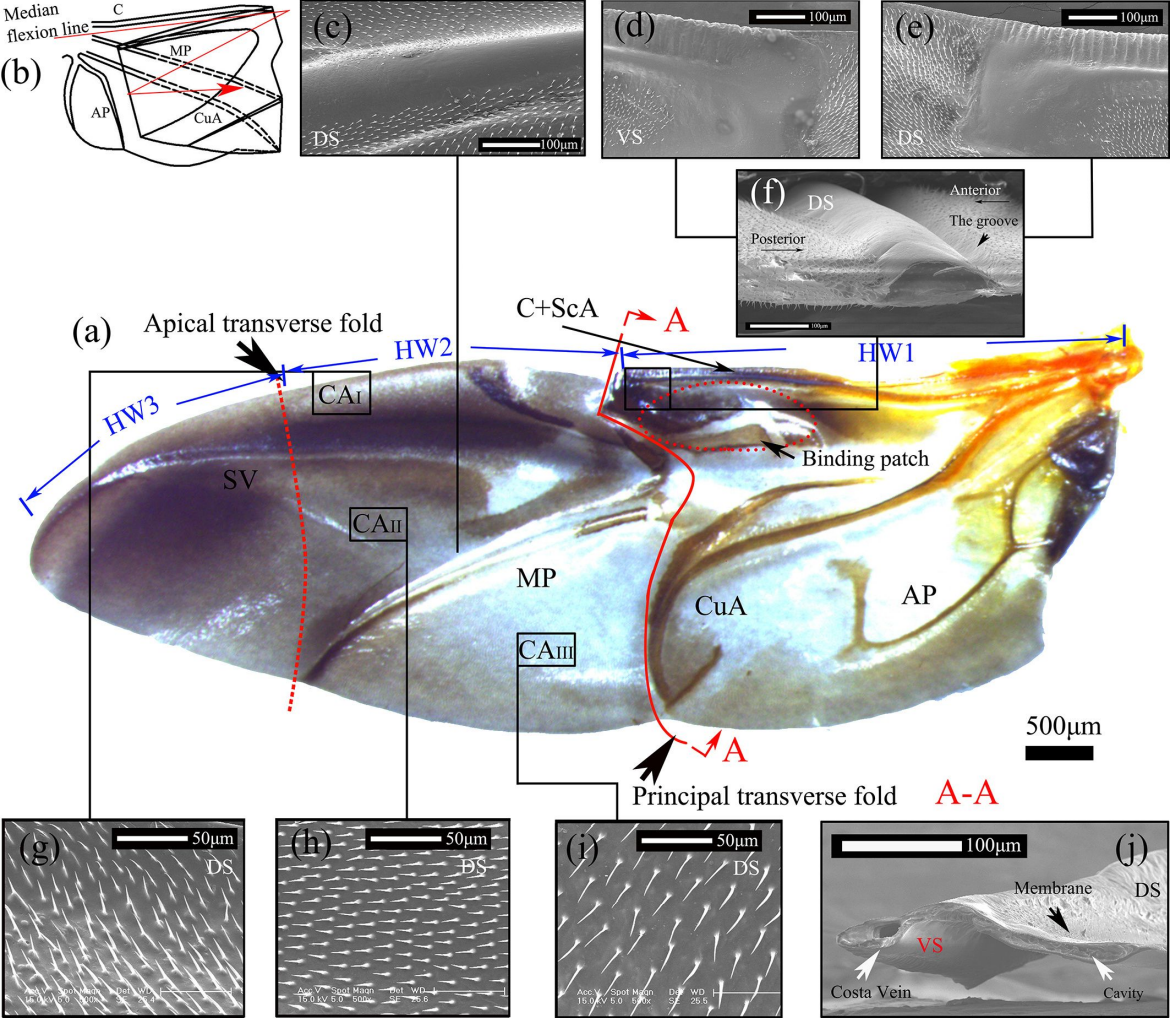
The DS of the hindwing of *H. axyridis* in an unfolded state (a) and a folded state (b). (c) shows the detailed microstructure of the corrugated membrane. (e) and (d) show the microstructural details of the end of the costal vein and its DS and VS, respectively; the microtrichia were sparse around the fold line but were dense away from the fold line. They were regularly distributed in the (g) costal field, (h) claval field, and (i) vannal field. (f) and (j) show cross sections of the costal vein in the principal transverse fold. C, costa; ScA, subcosta anterior; MP, media posterior; CuA, cubitus anterior; AP, anal posterior; SV, supporting vein. CAI, CAII, and CAIII are the regions used for measuring CAs. HW1–3 represent different folding segments of the hindwings (HW).

The surface hydrophobicity was measured for both the dorsal side (DS) and the ventral side (VS). There are many veins in the wing base, resulting in an uneven surface that affects measurements. Therefore, we selected the posterior margin, which is relatively flat. The contact angles (CAs) of the three main fields of the posterior margin of the hindwing were measured, namely, contact angle testing regions I–III (CAI–CAIII) (Figure 2a), using the contact angle system (OCA20, Dataphysics Instruments GmbH, Germany), and tests were performed using the sessile drop method. The DS and VS of the hindwings were fixed on glass slides with AB glue. The volume of the water droplets was 0.05 μL.

## Results

### Microtrichia on the hindwing surface

Images of unfolding of the whole hindwing were captured using a stereomicroscope (Figure 2a), and a picture was generated of the hindwing folding into a Z-shape along the principal transverse fold and apical transverse fold (Figure 2b). Figure 2a shows the principal transverse fold on the center of the entire wing, which forms a curve. Moreover, the costal vein almost disappears at the principal transverse fold, and the joint at the end of the costal vein is larger and thicker.

Figure 2c–j show photographs obtained using ESEM, and Figure 2c–e show the hindwing surface microstructure of the DS and VS along the principal transverse fold. The images show that these microtrichia are sparse around the fold line and vein but are much denser away from the fold line. In particular, the microtrichia of the costal vein were the least dense, and there was a distinct dividing line at the fold. There was a difference between the anterior and posterior edges of the costal vein (C+ScA) on the DS. There was a small groove on the anterior side, but the posterior side was relatively flat (Figure 2f). This small groove was slender and smooth, with no microtrichia; however, it was not found on the VS.

Figure 2g–i show that microtrichia were regularly distributed. The direction of the anterior microtrichia was upward (Figure 2g); that of the middle microtrichia was horizontal and backward (Figure 2h); and that of the posterior microtrichia was downward (Figure 2i).

The cross-sectional thickness in the costal vein area showed a decreasing trend from base to tip. In general, the thickness decreased from 56.1 μm to 3.15 μm. The costal vein is a hollow elliptical structure that is similar to the blood vessels (Figure 2j) and is near a large cavity in the hindwing that could decrease weight and improve flexibility [22].

### Microtrichia on the ventral side of the elytra

To adapt to their environment, *H. axyridis* have evolved hindwings with a self-locking function, with microtrichia distributed in different directions. Our studies have demonstrated that hydraulic pressure can promote hindwing unfolding [9], but the mechanism underlying the folding and extension of the hindwings in *H. axyridis* required further investigation.

**Figure 3:**
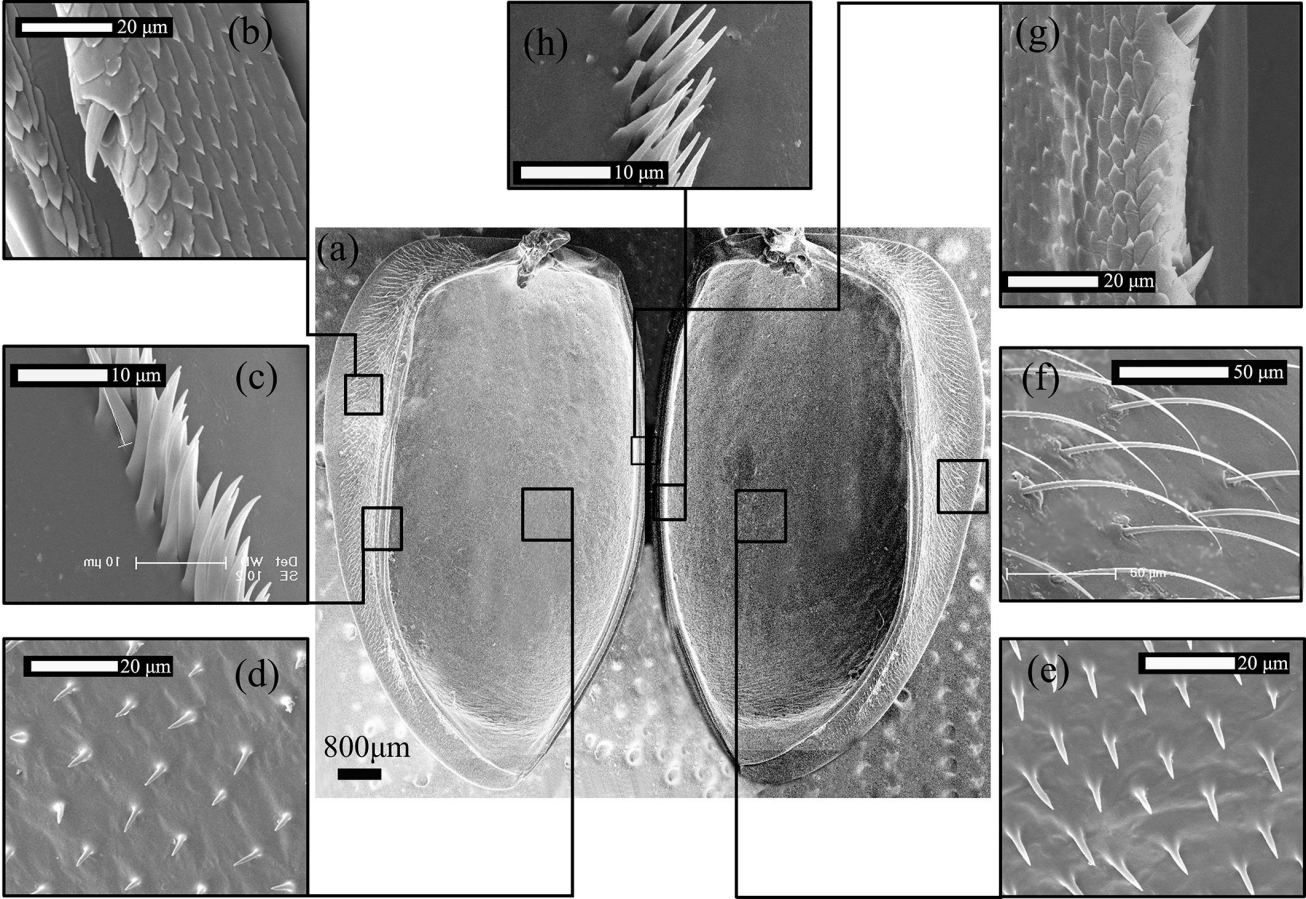
(a) A SEM photograph of the ventral side of *H. axyridis* elytra; (b, g) the second-level microtrichia and lateral binding patches on the elytra, respectively; (c, h) the third-level microtrichia and sutural binding patches on the elytra; (d, e) the fourth-level microtrichia and patches of small spicules; and (f) the first-level microtrichia with a patch of long setae.

The SEM photograph of the elytra on the VS (Figure 3a) shows ESEM images of microtrichia (Figure 3b–h) of different lengths and shapes (Table 1). It contains three different surfaces: the largest central part is the internal face of the elytron. The lateral edge is the epipleuron, tucked below the VS of the pterothorax and of the abdomen. Figure 3b, 3c, and 3f show the external surfaces. They have no contact with the trunk but may contact wings while packed in the folded position. The internal surface of the epipleuron comes in direct contact with the thorax, with the costal edge of the folded wing (HW1) and with the abdomen. The left and right sides of the internal face of the elytra were symmetrical. The 1st level, on the external surface of the epipleuron, is shown to have a patch of long setae in Figure 3f. It is bent sideways, but the exact direction is inconsistent. The 2nd level (Figure 3b and 3g) had cone-shaped microtrichia that were arranged in a straight line. They were arranged individually and in a loose pattern, with lateral binding patches on the elytra. As shown in Figure 3b, there is a longitudinal mesonotal groove that locks with similar patches on the abdomen [13]. The longitudinal mesonotal groove is the strongest lock of the elytra. In a live beetle, it is impossible to open the elytra without causing direct damage to the tucked antero-medial edge of the elytron. To lock this connection, the meso- and metaterga must be drawn together with a special muscle [8]. Its antagonist bends the mesotergum forward, loosening the lock. The second strongest lock is between the posterior edge of the pronotum and the anterior edge of the closed elytron. The groove can also hold the flexed inner edges of the elytra [23]. This capability may be necessary for the beetle to minimize water evaporation in dry climates [13]. Figure 3g shows that the most medial microtrichia were squamous and arranged in parallel. The microtrichia were tightly arrayed and pointed upward, and there were spikes on their outer side, which would improve elytral stability when both elytra are closed. In the folded wing, high-friction binding patches lie parallel to the longitudinal axis of the body in a central position beneath the elytral suture [5,24]. The 3rd level (Figure 3c and 3h), which had sutural binding patches on the elytra, exhibited microtrichia organized like a row of tightly bundled grass that grew straight upward in a dense arrangement. The direction of the comb in Figure 3c shows an unusual diagonal arrangement with respect to the epipleuron. In the 4th level (Figure 3d and 3e), which shows patches of small spicules, these microtrichia were arranged symmetrically around the central axis, and they were arranged in parallel and in the same direction in a manner suggesting that they were strongly involved in the self-locking function. Microtrichia situated on the elytral VS may play a role in holding the DS of the hindwing when abdominal movements push the more apical parts of the hindwing ventrally and anteriorly [5].

**Table 1:** Morphological parameters of microtrichia of elytra of *H. axyridis* on VS.

	Length, μm	Width, μm	Distance between microtrichia, μm	Morphology	Position	Figures

1st level	112.5–124.5	2.7–4.2	29.30 ± 0.56	long setae	external surface of epipleuron	Figure 3f
2nd level	16.7–19.2	5.6–6.9	50.01 ± 0.09	cone-like	external surface of epipleuron	Figure 3b
	4.6–7.7	2.1–3.5	2.84 ± 0.53	squamous	internal face	Figure 3g
3rd level	11.2–17.5	1.8–2.1	–	tightly bundled, grass-like	external surface of epipleuron	Figure 3c
	6.3–11.9	1.4–1.9	–	tightly bundled, grass-like	internal face	Figure 3h
4th level	5.6–8.4	1.4–2.1	11.37 ± 0.55	small spicules	internal face	Figure 3d,e

### Microtrichia of the abdominal surface

**Figure 4:**
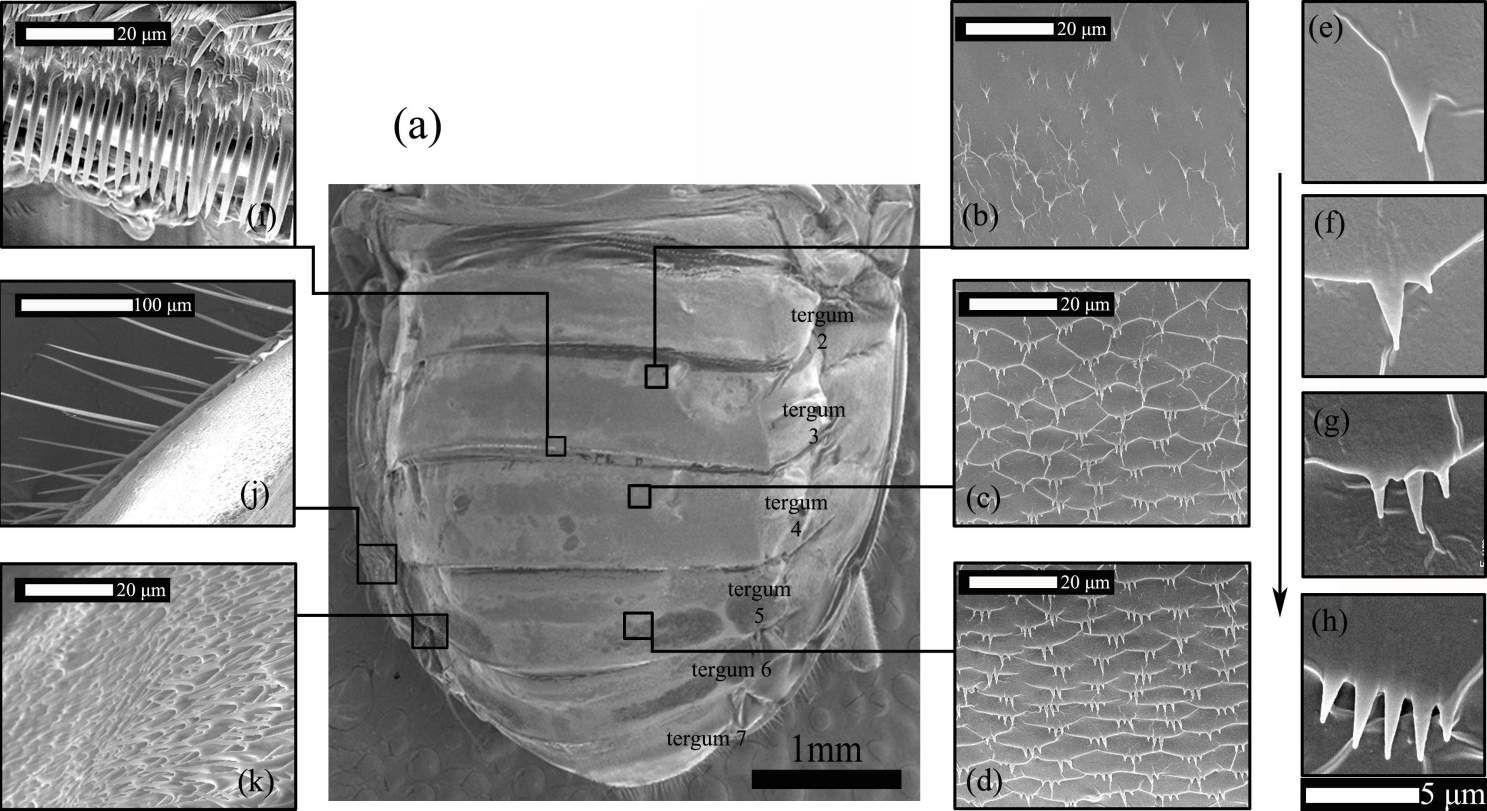
(a) SEM photograph of the abdominal terga of *H. axyridis*; (b, c, d) the pattern of microtrichial arrangement on the middle of the abdomen; (j, k) the microtrichia on both sides of the abdomen; (j) the lateral margin; (k) part of the spicule patch; (i) part of the palisade fringe at the posterior margin of the tergum; (e, f, g, h) the number of microtrichia in each cluster gradually increases from single to multiple in the middle of the abdomen.

Figure 4a shows a SEM photograph of the surfaces of the abdomen of *H. axyridis*. Overall, the abdomen was composed of six abdominal segments. ESEM imaging showed that in terga 3–5, the microtrichial arrangement exhibited cyclical changes in the middle of the abdomen. The density of the microtrichia changed from sparse to dense in each abdominal segment (shown in Figure 4b–d), and the number of microtrichia in each cluster gradually increased from single to multiple, corresponding to the change in density (Figure 4e–h). The spacing of these tergal, wing-folding spicules corresponds to the spacing of the wing-surface microtrichia with which they mesh during abdominal pushes. At the posterior margin of the tergum, the shape of the microtrichia changed dramatically, forming part of the palisade fringe, and they were perpendicular to the surface (Figure 4i). The lengths of the microtrichia were 14.9–20.5 μm, the widths were 2.3–2.7 μm, and the distance between them was 3.53 ± 0.03 μm. The microtrichia also function in grooming and cleaning behaviors when the hindwings are wet and dirty or during stroking movements of the abdominal apex [5].

On the lateral margin of the abdomen (Figure 4j), the microtrichia were slender, straight and parallel to each other. They were 90.2–175 μm long, 2.7–5.6 μm wide, and 102 ± 0.26 μm apart. Except for those in the fan-shaped region in the middle, the microtrichia were mostly longer than 14 μm and arranged densely and in the same direction, and they symmetrically covered both sides of the abdomen (Figure 4k).

Table 2 shows the angular orientation of microtrichia on the hindwing, elytron and abdomen in *H. axyridis*, including the angle with respect to the horizontal (tangent) plane. The angular orientation was measured for approximately 20–30 microtrichia in each position, and the mean values were used.

**Table 2:** Angular orientation of microtrichia on the hindwing, elytron and abdomen of *H. axyridis*.

Parts	Locations	Horizontal angle/°

Hindwing (Figure 2)	g	55.33 ± 6.81
	h	3.33 ± 2.52
	i	51.67 ± 1.53

Hindwing (VS)	HW1	73.33 ± 4.73
	HW2	42.67 ± 10.29
	HW3	43.33 ± 14.94

Hindwing (DS)	HW1	44.64 ± 7.88
	HW2	57.50 ± 8.50
	HW3	5.75 ± 1.54

Elytron (Figure 3)	b	80.01 ± 1.48
	c	76.50 ± 2.12
	d	65.67 ± 5.13
	e	70.33 ± 0.58
	f	21.50 ± 7.78
	g	53.00 ± 1.41
	h	49.33 ± 4.16

Abdomen (Figure 4)	b	84.67 ± 8.39
	c	85.00 ± 2.65
	d	83.00 ± 4.36
	i	11.67 ± 1.53
	j	13.33 ± 1.53
	k	8.67 ± 4.93

### Contact angle of the hindwings

**Figure 5:**
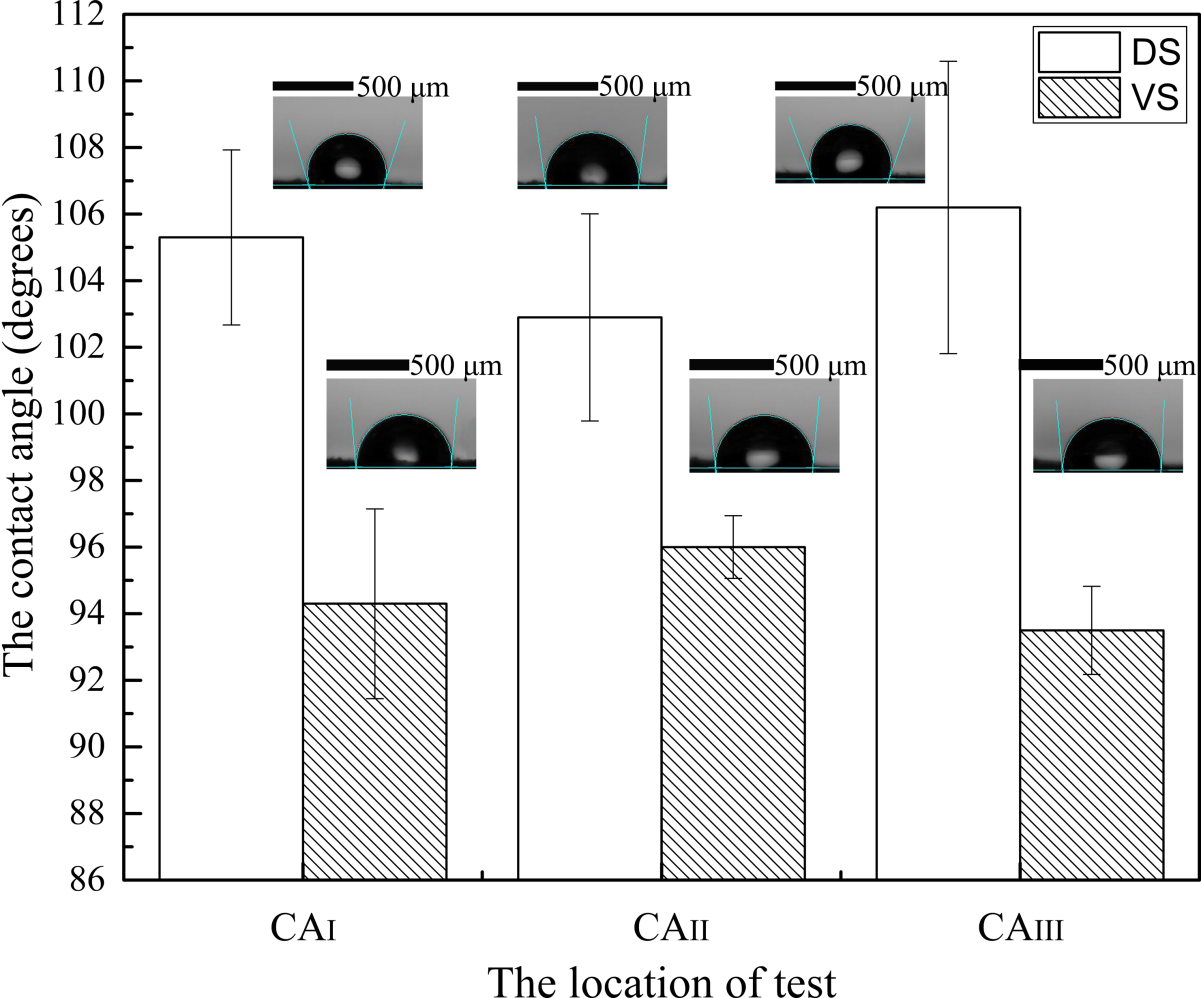
The contact angles for CAI, CAII, and CAIII for *H. axyridis* hindwings.

To further understand the hydrophobic function of *H. axyridis* hindwings, a standard-type contact angle meter was used to quantify their anti-wetting function. The regions that were measured included CAI, CAII, and CAIII (in the hindwings), and it can be seen from Figure 5 that the CAs of the DS and VS were greater than 90°. The CA values for CAI, CAII, and CAIII were 105.33 ± 2.63°, 102.93 ± 3.11° and 106.20 ± 4.39° on the DS and 94.28 ± 2.85°, 95.98 ± 0.94° and 93.59 ± 1.32° on the VS, respectively. This conformation shows that the hindwings of *H. axyridis* are hydrophobic. However, the CAs of the DS were generally greater than those of the VS.

### *H. axyridis* hindwing-folding mechanism

**Figure 6:**
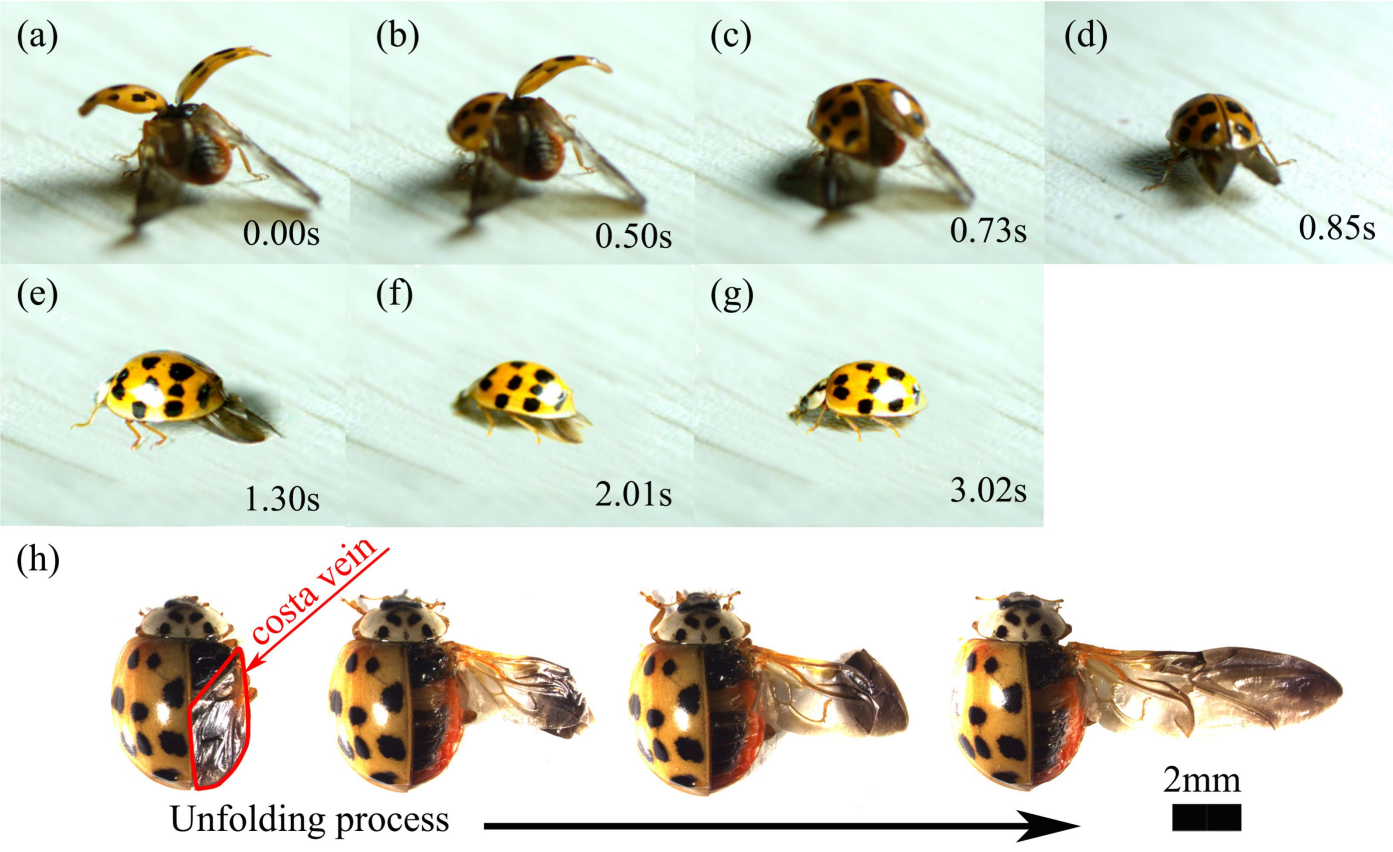
The hindwing folding and unfolding processes of *H. axyridis*. (a–g) Dynamic views of folding acquired with a high-speed camera and (h) static views of unfolding actions acquired with a stereomicroscope.

During evolution, microtrichia have taken different forms on the elytra, hindwings and abdomen. For the function of microtrichia, these different forms all play an important role in the hindwing-interlocking mechanism. The hindwings of *H. axyridis* can freely fold into the elytra, and they will not slide out from the elytra while the animal is crawling. This phenomenon is highly dependent on the microtrichia located on the surfaces of the elytra, hindwings and abdomen. The folding of *H. axyridis* hindwings was captured using a high-speed camera (Figure 6a–g), and Figure 6h shows the unfolding of hindwings.

Figure 6a–g show the folding of *H. axyridis* hindwings. After *H. axyridis* landed, the opened elytra were gradually returned to their original pattern in an orderly manner. Then, the hindwings were retracted and finally hidden under the elytra. Because the elytra and abdomen are curved, the folded hindwings exhibit passive bending and stick in the middle, where the DS and VS make direct contact with the elytra and abdomen, respectively. With the contraction (sometimes called “pumping” [25]) of the abdomen, *H. axyridis* hindwings were retracted under the elytra in a stepwise fashion (Figure 6d–g). To determine the movements by which folding is achieved, the right elytron was removed from a live *H. axyridis*, and its hindwing was expanded step by step according to lines made by its folding (Figure 6h).

## Discussion

The different distribution patterns of microtrichia may be related to their different functions. One function is to provide a series of interlocking structures. These structures enable the hindwings of *H. axyridis* to lock after folding due to interactions with the elytra and abdomen, and they can maintain a static state [26]. Based on the direction in which they point (which varies within a given wing), the microtrichia on the hindwings on the DS could mesh with abdominal microtrichia [5]. A second function is air deflection in flight. When air flows over the surface of the hindwings, the microtrichia tilt because of the force of the wind, which can increase the time the boundary layer contacts the air, promoting laminar flow and preventing the formation of turbulent eddies [27]. The microtrichia also function as anti-wetting structures [28]. Previous studies have shown that the anti-wetting function of microtrichia enables flying insects to easily overcome difficulties caused by getting wet during flight.

The results presented here indicate that the anti-wetting effect of the DS is stronger than that of the VS. The interfacial properties and functional efficiency of the DS were superior. On the other hand, for both the DS and VS, the differences in values were small between the three main fields (CAI, CAII, and CAIII). These results demonstrate that the level of hydrophobicity was almost equivalent between the partitions. That is, the functionality of the three fields was equal. Studies have shown that the average CA of the cicada wing surface is 150° [25]. Thus, *H. axyridis* hindwings meet the criterion for hydrophobicity (CA ≥ 90°). Some studies of dragonfly and damselfly wings have demonstrated that CAs are in the range of 120–136° [29]. The results of these studies demonstrate that insect wings have hydrophobic activities. Some insects can perform normal flapping flight in the rain, and their wings are kept dry, allowing them to contend with environmental risks [30–33]. In addition, dirt on insect wings may increase wind resistance and energy consumption [34], and the hydrophobic structure of the microtrichia may also prevent dust intrusion so that the wings can be kept clean, thereby improving flight efficiency. More importantly, microtrichia maintain space between overlying fields of the wing and prevent cohesion caused by the adsorbed water. By measuring the water CA of dragonfly and damselfly wings with a standard contact angle meter, researchers found that the CA varied in the range of 120–136° for both species when wings were moved from the basal to the distal region [29]. This configuration explains why almost all dragonflies can fly in the rain without getting wet [35]. Additionally, butterfly wings exhibit excellent hydrophobic characteristics; water drops can move freely on the wing surface [36]. Through bionic design, many artificial hydrophobic structures imitating the microtrichia of insects have been successfully manufactured.

**Figure 7:**
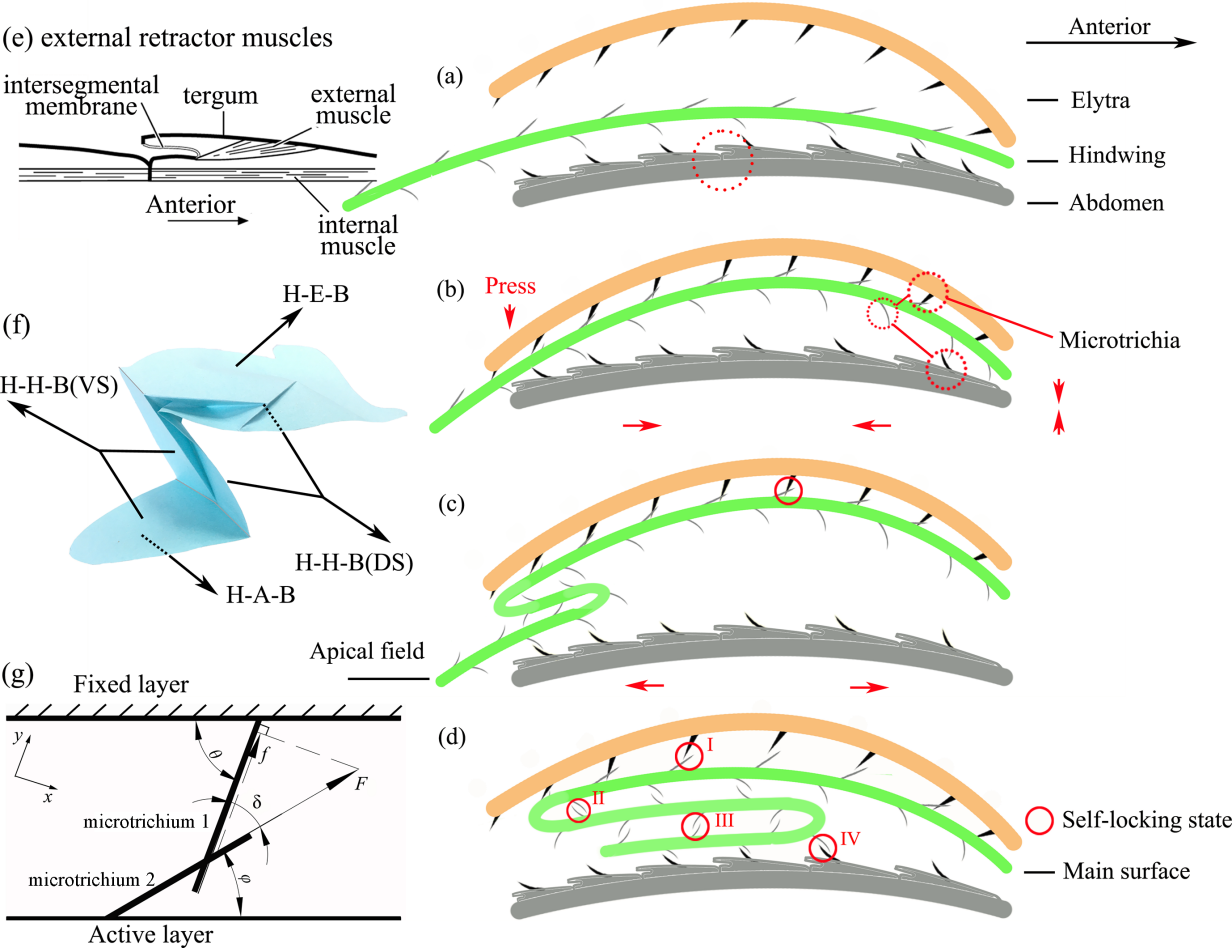
The interlocking model of hindwings of the *H. axyridis.* (a–d) show the interlocking model of a *H. axyridis* hindwing corresponding to Figure 6a–g. (e) Diagram of the dorsal longitudinal musculature in an abdominal segment and the typical arrangement of external and internal muscles, both of which act as retractors [27]. (f) Schematic diagram of a folded hindwing. H-E-B is the binding of hindwing and elytra; H-H-B (DS) is the binding of the hindwing to itself on the DS; H-H-B (VS) is the binding of hindwing to itself on the VS; H-A-B is the binding between the hindwing and the abdomen. (g) Schematic of interaction forces of microtrichia during folding process.

A model was established based on the folding of the hindwings and the microstructural characteristics of the VS of the elytra, the DS and VS of the hindwings, and the surface of the abdomen (Figure 7a–d, viewed from the right side of the *H. axyridis*). The elytra, hindwings and abdomen are simplified in the different colorized curves. Typically, the abdominal terga show secondary segmentation, with the anterior part of a segment overlapping the posterior part of the segment above it. When moving through tight spaces, this overlap protects the beetles from injury [37]. The various types of microtrichia are simplified as binding and thorn-like shapes. The model was established based on three sections: the VS of the elytron, hindwings and abdomen. The second section (hindwing) is more complex because it changes during the folding process. The folding process can be divided into three steps as follows.

The first step is the initial state and is shown in Figure 7a, which corresponds to Figure 6b. At this time, the elytra fall, and the hindwings, which are controlled by the wing basal muscle, rotate 90° and fold down. The elytra, hindwings and abdomen are not in contact. The hindwings are still in the unfolded state, and abdominal contraction has not begun.

The second step is shown in Figure 7b, which corresponds to Figure 6c. The elytra press the hindwings to fold against the abdomen, as shown by the red arrows. The VS of the elytra directly contacts the DS of the HW1 section of the hindwing. As shown in Table 2, the directional orientation of microtrichia on the internal face of the elytron is 70.33 ± 0.58° in the horizontal direction; correspondingly, the directional orientation of microtrichia in the region of the hindwings is 44.64 ± 7.88°. The microtrichial orientations between the VS of the elytra and the DS of the hindwing are opposite, which make them bind together. This demonstrates that the structures that result in elytron/wing binding based on microtrichia enable the wings to maintain a proper angle and achieve the correct sequence of folds through abdominal pushing. Immobilization of a folded wing is often due to close contact between two patches of high-friction binding spicules, one in the wing and the other in the elytron [28]. At this time, the elytra and hind wings stick together and have no contact with the abdomen. The two elytra open at the same time; however, they close in turn. The folding distance of the relatively flexible trailing edge of the hindwing is short [5].

The third step is shown in Figure 7c,d, which correspond to Figure 6d–g. The elytra are close together, and the VSs of the hindwings (HW2 and HW3) make direct contact with the abdomen. The posterior margins of the hindwings do not overlap, and they align along the sides of the suture and stick to the binding patches, which bind with terga 2–4. HW1 on the DS is stuck on the elytra. In this closed space formed by the elytra and abdomen, the interaction of elytra, hindwings and abdomen is still in progress. When the abdominal segment extends under the combined effect muscles and the curvature of the abdomen, these structures overlap and move together, and the wings (the VSs of HW2 and HW3) easily slide along the abdomen. At this time, the VSs of HW1 and HW2 bind to each other. The directional orientations of microtrichia on the VSs of HW1 and HW2 are 73.33 ± 4.73° and 42.67 ± 10.29° in the horizontal direction, respectively, which is similar to those on the elytra and the DS of HW1. The microtrichia on parts of HW1 and HW2 bind together, providing high friction that prevents the folded hindwings from slipping out of the elytra. Additionally, the DSs of HW2 and HW3 bind together. The directional orientations of their microtrichia are 57.50 ± 8.50° and 5.75 ± 1.54° in the horizontal direction, respectively, which are very different from those in the HW1-elytron and HW1–HW2 (VS) interactions. The intersection of these two sets of microtrichia allows them to more easily slip past each other, which is consistent with CA test results showing that this region is hydrophobic because of the microtrichia. This can also prevent cohesion of layers when they are folded under the elytra. Then, when the abdominal segment contracts, the apex of the tergum rises and tilts rearward, acting like a ratchet because the microtrichia have fallen into the “dip” between the terga and the hindwings and therefore cannot slip out of the elytra, which means that the tergum will carry the elytra with the hindwings tucked inside. The intersegmental membrane can be stretched and shortened according to working conditions. The directional orientations of microtrichia in HW3 on the VS and abdomen are 43.33 ± 14.94° and 83.00 ± 4.36°–85.00 ± 2.65° in the horizontal direction, respectively, which are similar to those of microtrichia of the HW1-elytron and HW1–HW2 (VS) interactions. It was demonstrated that directionality in microtrichial fields contributes to their ability to move in functionally beneficial directions, some for high-friction binding and some for the easy slipping of sections. Because the density of the microtrichia on one abdominal segment ranges from sparse to dense (Figure 4e–h), especially the palisade fringe at the posterior margin of the tergum (Figure 4i), when an abdominal segment extends again, HW2 will not be moved away, and the microtrichia will act as a braking device. The folding results from the structure and elasticity of the cuticles of the veins, which are discontinuous between the proximal and distal parts of the veins in Coleoptera [23]. An investigation of wing folding in *Allomyrina dichotoma* found that microtrichia on the body surface can improve the folding process [38]. With stroking movements of the abdominal apex, HW2 and HW3 of the hindwings are slowly folded into a Z-shape. Retraction of the hindwings is accompanied by a stroking movement of the abdomen, in which the internal muscles can retract the interlocking segments and the external muscles are posterior to the insertions [23]. The abdomen seems to tense the springs of the costal and cubital bars during folding for the wing to unfold automatically [28]. An investigation of *Coccinella septempunctata* (L.) observed multiple abdominal lifting movements [14].

Because the abdomen is curved, the length of HW2 is equal to that of terga 5–7; the microtrichia beyond tergum 4 serve as obstacles that participate in positioning parts of the wing during folding, ensuring a correct sequence of folds and retains the wing in a fully folded condition. To overcome elasticity these simple transverse folds are usually opened to the extended position and rely on “preening” movements of the abdomen [39]. At this point, the folding is basically completed, and the final state has been achieved. In this state, the main surface of the hindwings and the elytra form a self-locking state, and the apical field of the hindwings and the abdomen form a self-locking state. Setose binding patches on the inner elytral surface, hindwing surface, and abdominal terga are used in addition to the wing folding characteristics, to complete the wing folding. This ensures that the hindwing membranes are safely stowed [37]. The hindwings fold into a Z-shape and also form a self-locking system. The microtrichia of the DS and VS bind to each other, making the wings more stable in the enclosed space. The folded state will be maintained, and the hindwings will not slide out of the enclosed space when a disturbance occurs on the outside. In addition to helping retract the hindwings, the abdomen also moves along with the elytra during breathing and movement. Why do the hindwings not slip outside? When the elytra lift, the hindwings are easily lifted along with the elytra, which spread actively at this moment due to the cooperative activity of meso- and metathoracic muscles [8]. In addition, the unfolding time of hind wings (0.24–0.55 s) is shorter than that of the folding time (≈3 s). Therefore, it can be deduced that the friction force of the DS–DS and VS–VS interactions is greater than that of the interaction between the VS and the abdomen. An additional explanation is that the vein is like a spring-loaded tape measure (that is, a carpenter’s tape) that can stabilize in the unfolded shape and confer sufficient stiffness for flight [14]. In addition, during repeated hindwing folding, the resilin in wing folds has an important role in preventing material damage to hindwings [10].

A force schematic (Figure 7g) of the folding process was established and can be used to explain the interaction forces between microtrichia. In Figure 7g, the upper layer is fixed, and the lower layer is moved relative to it (active layer). The angle between the fixed layer and its microtrichia are marked as θ; the angle between the active layer and its microtrichia is marked as φ. The intersection angle of two microtrichia is marked as δ, where δ = θ − φ. During folding, microtrichium 2 of the lower layer always slides toward the upper layer. The force (*F*) is what makes the lower layer close up to the upper layer. The force acting in the direction of microtrichium 1 is its component force (*f*), which is the friction between two microtrichia, and its direction is upwards along the microtrichia, calculated as *f* = *F*·cosδ. According to the angle data in Table 2, it can be concluded that the δ at positions I, III and IV (Figure 7d) are approximately 30°, and the angle of position II is approximately 50°. According to *f* = *F*·cosδ, *f* at positions I, III, and IV is greater than at position II, which means that the binding between microtrichia at positions I, III, and IV is strong and stable. In contrast, binding at position II is less stable, and the interactions are not as strong. This is because this position moves and changes during the entire process of folding the hindwing, and less friction allows more movement of one layer against another layer.

In summary, the interlocking mechanism of the hindwings of *H. axyridis*, which can keep their hindwings folded without slipping out of the elytra, includes self-locking of the elytra, high friction between the microtrichia of the DS and VS, and the ratcheting of microtrichia on the DS and the abdominal terga. Additionally, the directional orientation of microtrichia plays an important role in hindwing folding.

## Conclusion

To investigate the role of microtrichia in *H. axyridis* in the mechanism underlying the interlocking of the hindwing, the microstructures of microtrichia on the elytra, hindwing and abdomen were observed using ESEM. Then, the different functions of microtrichia were discussed. On the hindwing, there are fewer microtrichia around the fold line and more microtrichia in places farther from the fold line. Additionally, these microtrichia are symmetrically distributed. The structures of the left and right VSs of elytra are symmetrical, and there is a transitional structure from the lateral edge to the medial edge. Microtrichia in different locations have different morphologies and functions. The arrangement of microtrichia on the abdominal terga exhibits a cyclical change in the middle of the abdomen. The density of the microtrichia shifts from sparse to dense in each abdominal segment, and the number of microtrichia in each cluster gradually increased from single to multiple corresponding to the change in density. Based on observations of the hindwings, elytra, and abdomen, the interlocking model was established. On the basis of measurements of the directional orientation of microtrichia, it was demonstrated that directionality in microtrichial fields contributes to their ability to move in functionally beneficial directions. In addition, the CA experiment results showed that the CAs of the regions CAI, CAII, and CAIII on the DS were larger than those of the same regions on the VS, and all CAs were greater than 90°. Additional functions of microtrichia include not only hydrophobicity but also preventing cohesion of the folded hindwing layers, favoring quick unfolding of the hindwings. This basic investigation will be beneficial for understanding the wing-folding/unfolding mechanisms in *H. axyridis*.

## References

[1] Huda N, Anwer S F, Saha A K, Das D, Srivastaja R (2017). The effects of leading edge orientation on the aerodynamic performance of dragon fly wing section in gliding flight. 5th International and 41st National Conference on Fluid Mechanics and Fluid Power.

[2] Saito K, Okabe Y Elastic Wing Deployments in Beetles and Their Folding Mechanisms. Proceedings of the ASME 2015 International Design Engineering Technical Conferences and Computers and Information in Engineering Conference.

[3] Hammond P M (1985). Biol J Linn Soc.

[4] Gorb S N (2001). Am Zool.

[5] Hammond P M, Ervin T L, Ball G E, Whitehead D R (1979). Wing-folding Mechanisms of Beetles, with Special Reference to Investigations of Adephagan Phylogeny (Coleoptera). Carabid Beetles.

[6] Karner M, Blickhan R (1997). Bifunctional muscles and interlocking mechanism in cantharis: How not to move the wings. Abstract Papers II. Biomechanic Workshop of the studygroup morphology.

[7] Kukalová-Peck J (1983). Can J Zool.

[8] Frantsevich L (2010). J Exp Biol.

[9] Sun J, Ling M, Wu W, Bhushan B, Tong J (2014). Int J Mol Sci.

[10] Haas F, Gorb S, Blickhan R (2000). Proc R Soc London, Ser B.

[11] Michels J, Appel E, Gorb S N (2016). Beilstein J Nanotechnol.

[12] Hass F, Beutel R G (2001). Zoology (Munich, Ger).

[13] Fedorenko D N (2009). Evolution of the Beetle Hind Wing, With Special Reference to Folding (Insecta, Coleoptera).

[14] Saito K, Nomura S, Yamamoto S, Niyama R, Okabe Y (2017). Proc Natl Acad Sci U S A.

[15] Frantsevich L (2012). Zoology (Munich, Ger).

[16] Frantsevich L, Dai Z, Wang W Y, Zhang Y (2005). J Exp Biol.

[17] Gorb S N (1998). Int J Insect Morphol Embryol.

[18] Samuelson G A, Furth D G (1994). An elytron to body meshing mechanism of possible significance in the higher classification of Chrysomelidae (Coleoptera). Proceedings of the Third International Symposium on the Chrysomelidae.

[19] Samuelson G A, Jolovet P H A, Cox M L (1996). Binding sites: Elytron-to-body meshing structures of possible significance in the higher classification of Chrysomeloidea. Chrysomelidae Biology: The Classification, Phylogeny and Genetics.

[20] Burks R A, Heraty J M (2015). Arthropod Struct Dev.

[21] Qian J, Chi D, Chai R (2016). J For Res (Harbin, China).

[22] Xiang J, Du J, Li D, Zhen C (2016). Microsc Res Tech.

[23] Chapman R F (1998). The Insects Structure and Function.

[24] Frantsevich L (2012). J Insect Physiol.

[25] Hu H-M, Watson J A, Cribb B W, Watson G S (2011). Biofouling.

[26] Linghu Z, Zhao C, Yang H, Zheng X (2015). Sci Bull.

[27] Hu H-M, Watson G S, Cribb B W, Watson J A (2011). J Exp Biol.

[28] Fedorenko D N (2015). Biol Bull Rev.

[29] Aideo S N, Mohanta D (2016). Appl Surf Sci.

[30] Sun T, Feng L, Gao X, Jiang L (2005). Acc Chem Res.

[31] Gorb S N, Kesel A, Berger J (2000). Arthropod Struct Dev.

[32] Gao X, Jiang L (2004). Nature.

[33] Sun M, Watson G S, Zheng Y, Watson J A, Liang A (2009). J Exp Biol.

[34] Watson G S, Cribb B W, Watson J A (2010). J Struct Biol.

[35] Krishnan K G, Milionis A, Loth E, Farrell T E, Crouch J D, Berry D H (2017). Appl Surf Sci.

[36] Bormashenko E, Bormashenko Y, Stein T, Whyman G, Bormashenko E (2007). J Colloid Interface Sci.

[37] Resh V H, Cardé R T (2003). Encyclopedia of Insects.

[38] Murphy J T, Hu H (2010). Exp Fluids.

[39] Brackenbury J H (1998). Wing Folding in Beetles.

